# Making peace with disliked others: the effects of a short loving-kindness meditation on implicit and explicit emotional evaluations

**DOI:** 10.1186/s40359-022-00817-5

**Published:** 2022-04-29

**Authors:** Franziska Anna Schroter, Petra Jansen

**Affiliations:** Department of Human Sciences, University of Regensburg, Regensburg, Germany

**Keywords:** Loving-kindness meditation, Imagery, Affective priming, Implicit attitudes, Explicit attitudes

## Abstract

**Background:**

The main goal of the study was to investigate the effects of a short loving-kindness meditation (LKM) on explicit and implicit evaluations of oneself and disliked public persons. We expected a more positive explicit and implicit evaluation of oneself and a disliked public person after the LKM and a mood improvement.

**Methods:**

Before and after the implementation of a short LKM vs. imagery task, mood, explicit and implicit evaluations were analyzed in 69 students.

**Results:**

Our results demonstrated only a reduction in negative and positive mood in both groups and regarding the explicit and implicit tasks, only a significant main effect of picture and a trend for the time*group interaction for mood, implicit and explicit attitudes with medium effect-sizes.

**Conclusions:**

A possible influence of a short intervention on emotional evaluations should be treated with caution. The claim that a short loving-kindness meditation enhances social connectedness might awake false hopes. This study suggests being careful with the interpretation of single meditation effects and future studies should examine the effects of a long-lasting meditation training on explicit and implicit evaluations of the self and disliked politicians as well as the sustainability of those effects.

## Background

Politicians are public persons and the implicit and explicit attitudes toward them predict political voting behavior [1]. While explicit attitudes are those that people are aware of, implicit attitudes are those that people are not explicitly aware of and where activation cannot be controlled [2]. Specifically, implicit attitudes seem to be successful in predicting the behavior of undecided voters [3]. Both types of attitudes can be described by dual-process or dual-system models, which propose that human behavior has always controlled (or conscious) and uncontrolled (unconscious) aspects [4]. The controlled aspects can be measured with explicit, the uncontrolled ones with implicit measurements. Even though the correlations between both types of attitudes were only small, they are both valid tools to gain insight in the evaluations of people, groups and specific concepts [5, 6]. However, implicit attitudes are not better or more accurate than explicit attitudes. Accordingly, a more holistic comprehension of attitudes is possible when applying both measurements in the investigation of attitudes toward people, groups or e.g., sustainable behavior. Given that these judgments can influence our behavior, it would be interesting to investigate if those explicit and implicit evaluations can be changed by an appropriate intervention.

This question has already been explored by Hutcherson et al. [7]. They investigated if a brief loving-kindness meditation exercise increases social connection toward strangers. Unfortunately, there is no broad agreement on the different aspects of mindfulness and no single definition of mindfulness [8, 9]. According to Hölzel et al. [10] attention regulation, body awareness, emotion regulation and change in perspective of the self are the mechanisms behind mindfulness. On the basis of the two dimensions “activation” and “amount of body orientation”, Matko and Sedlmeier [11] developed a classification system with the following seven clusters of meditation forms: mindful observation, body-centered meditation, visual concentration, contemplation, affect-centered meditation, mantra meditation, and meditation with movement. Another differentiation is the one in attentional, constructive and deconstructive meditation practices [12]. In general, loving-kindness meditation (LKM) is a widely used form of meditation [13], which belongs to the constructive family of meditation [12] or the affect-centred meditation [11]. While attentional meditation practice is primarily concerned with attentional processes, the deconstructive family of meditation forms is rather characterized by insights into self-related processes, such as conscious experience. Relation and values orientation are important qualities of the constructive family of meditation [12]. In LKM, the focus lies on developing love for oneself, a beloved person, a stranger, and a person one does not like much. Beginning with a short sequence of focusing on the breath, the meditating person is instructed to formulate four sentences for the own well-being, as for example: “May I be peaceful, may I be happy, may I be safe, may I be loved”. Subsequently, the sentences are repeated for a loved person, a more neutral person, and a person, with whom some difficulties have arisen in the past [11, 14]. It was assumed that LKM, with its focus on warm-heartedness, would increase the development of positive emotions and emotional wellbeing of people practicing meditation for the first time more than the application of an attention based meditation for beginning meditators [15]. However, core meditations such as LKM, entail effort and can lead to physiological arousal [16]. It was also shown that the body scan might strengthen the ability to regulate emotions more than a breathing and a loving-kindness meditation [17].

In the study of Hutcherson et al. [7], participants were randomly assigned to either a loving-kindness meditation or an imagery condition. Before and after these interventions, the mood was assessed with the positive and negative affect scale (PANAS [18]), as well as the implicit and explicit evaluative responses to photographs of the participants themselves, a close other, as well as three neutral strangers. To control for non-specific aspects, the authors included the evaluative responses to a non-social object (a picture of a lamp). For the explicit rating, the participants had to answer on a 7-point Likert scale how connected, similar, and positive they felt toward the subject in the picture. For the implicit rating, an affective priming task was used. In the short loving-kindness meditation, participants were asked to imagine beloved people, like family or friends. In the next step, they were asked to open their eyes and look at the photograph of a neutral person. They were instructed to send the love to the unknown person in the photo (target person). Subsequently, they were asked to repeat the following sentence: “May you be well, may you be happy, may you be free from all physical and mental pain”. In the imagery condition, participants should imagine a neutral person with whom they do not associate a particular feeling. They were instructed to recall every detail of this person, from the shoes to the face. The results of Hutcherson et al. [7] showed that both groups had a more explicit and implicit positive evaluation of the target after the loving-kindness meditation but not following the imagery condition. This indicates that even after just a few minutes of loving-kindness meditation, the feelings of social connection and positivity toward novel individuals on both implicit and explicit tasks were ameliorated. A short bout of LKM might therefore be able to improve positive social emotions and even decrease social isolation [7]. The explicit ratings did not account for the increase in implicit positivity.

Most of the studies in mindfulness research investigate varying effects after several weeks of practicing. Only a few studies examined the influence of a single session of mindfulness on different outcomes. For example, it has been demonstrated that a 10-min meditation can improve executive functions, with the kind of improvement depending on the specific tests of executive functions [19]. Hutcherson et al. [7] state that it remains an open question if a short loving-kindness meditation can be used not only to develop positive emotions toward neutral strangers but also toward people who are disliked, which is the fourth step in the loving-kindness meditation. Accordingly, we aim to investigate if a short LKM intervention might also be helpful in improving the evaluations toward rather disliked people. We chose politicians as targets because they are a group of people who are often seen very critically and negatively on the one side, but on the other side have a high indirect influence on the well-being of the individual. Until now, only the development of stereotypes regarding politicians of specific subgroups, such as female [20] or black politicians [21], has been investigated.

Hence, we examined the efficacy of a short, guided loving-kindness visualization to increase positivity toward oneself, a stranger, three different disliked politicians and toward an object. In an adjusted replication of the study of Hutcherson et al. [7], the following hypotheses will be investigated:

1. Based on the findings of Hutcherson et al. [7] that the loving-kindness meditation led to a stronger increase in explicit positivity toward unknown strangers compared to the control group, we expect this effect to be present in our study, as well. Besides, we hypothesize that this effect will also apply to the explicit evaluations of disliked persons.
2. According to the results of Hutcherson et al. [7], a loving-kindness meditation, but not the imagery condition, led to an enhanced implicit positive response toward oneself. Because of the result that a loving-kindness meditation also led to an enhanced implicit positivity toward unknown strangers, we assume that the loving-kindness meditation, but not the imagery condition, will lead to an enhanced implicit positivity toward a disliked person, as well.
3. We also expect to see an improvement in the mood of the participants, as in the original study of Hutcherson et al. [7].

## Methods

### Participants

In the study of Hutcherson et al. [7], there was a significant three-way interaction of picture*group*time, with *F* = 2.42 in the explicit task. Based on their findings, the necessary sample size to find the same three-way interaction in our study with a power of 0.8 and an alpha value of 0.05 was estimated using BUCSS package for R and resulted in a required N = 76 [22]. This three-way interaction was also found for the implicit task in the study of Hutcherson et al. [7], with F = 2.31. Accordingly, a necessary sample size of 82 was estimated. Based on this estimation, 82 participants were recruited. 13 participants with 50% or more missing trials or false responses in the implicit task were excluded from the analysis. Overall, the sample population (*N* = 69) consisted of 38 female students (age: *M* = 21.87, *SD* = 2.37) and 31 male students (age: *M* = 22.23, *SD* = 1.98) attending the course “applied movement sciences” (a combination of sports science, psychology, and medicine). The age of the participants did not differ between groups, (age Imagery: *M* = 22.00, *SD* = 2.32, age LKM: *M* = 22.06, *SD* = 2.09, *t*(67) =  − 0.11, *p* = 0.910). The students were recruited via an online newsletter from the institute. Average meditation practice for the whole sample was 33.75 min per month (*SD* = 109.69) and 39.13% indicated to meditate on a regular basis, while 10.15% have never meditated and 50.72% only tried it once. Groups did not differ regarding monthly meditation practice, as demonstrated by a Wilcoxon-Mann–Whitney test: *Z* = 0.98, *p* = 0.326. Participants were assigned to the groups by block randomization and according to their time of participation. Participants were unaware of their group assignment as they were instructed to listen to a focusing exercise.

All participants received an information sheet in advance and signed the written consent declaration. After study completion, participants were informed about the background of the affective priming paradigm and the different experimental conditions. The experiment was conducted according to the ethical guidelines of the Helsinki Declaration and approved by the ethics board of the University of Regensburg (19-1619-101).

### Procedure

In advance to the experiment, demographical data was surveyed, including previous experiences in meditation practices. The whole experiment was implemented using the program OpenSesame. It started with a political rating task, where subjects had to indicate their opinion on eight different politicians. For this purpose, different politicians were rated regarding the question of how much sympathy the participants feel for him or her. As stimuli for the politician sympathy rating, we selected pictures from the internet of different well-known politicians from various countries in front of a white background, chosen based on public politician rankings [23] and based on familiarity. The pictures of each participant’s three most disliked politicians were used for the remaining experiment. Accordingly, the pictures could vary among participants. Subsequently, the participant’s explicit judgment toward him-/herself, a neutral stranger, the target politician (the most disliked politician), two other disliked politicians, and a neutral object (a lamp) to control for non-specific aspects, were assessed. The picture of the neutral stranger was derived from the Amsterdam Dynamic Facial Expression Set (ADFES) [24]. The picture of the participant was taken right before the experiment in front of a white wall and with a neutral face expression, in the same style as the pictures from the ADFES data base.

Implicit ratings were assessed using an affective priming paradigm with the same six pictures as in the explicit rating condition. We added a short practice trial for the affective priming task with four pictures of unknown persons. Besides, the current mood of the participants was assessed with the PANAS.

Following the baseline assessment, the intervention was carried out. It was delivered via audio speakers and included a 6-min audio instruction, guided by an accredited mindfulness teacher with more than ten years of teaching experience. In the short loving-kindness meditation, participants were asked to imagine beloved people or friends. In the next step, they were asked to open their eyes and look at the photograph of the politician they have rated as most disliked. They were instructed to send their love to this person. Subsequently, they were asked to repeat the following sentence: “May you be well, may you be happy, may you be free from all physical and mental pain”. Similarly, the control group heard the instructions of an imagery task with the same length as the LKM meditation and spoken by the same instructor. Those participants should imagine a person with whom they don’t associate a particular feeling. They were instructed to recall every detail of this person, e.g., his/her clothes. After four minutes the participants were asked to open their eyes and look at a photograph of the target politician and to focus on this person’s clothes. For both groups, a German translation of the instructions of the study of Hutcherson et al. [7] was used in order to achieve adequate comparability.

**Fig. 1:**
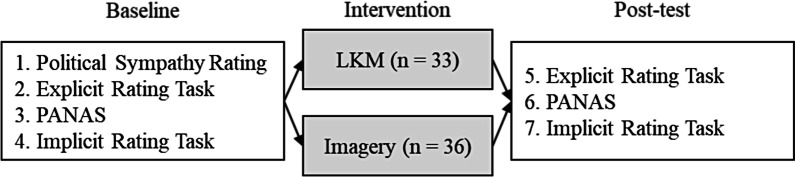
Experimental set-up. Allocation to the intervention groups was randomized

The mood rating, the explicit and implicit tasks were repeated after the intervention to gain insights into the changes evoked by the meditation. For a detailed overview on the order of the tasks see Fig. 1.

### Measures

#### Politician sympathy rating task

For the politician sympathy rating task, participants were asked: “How much sympathy do you feel regarding the person presented on the screen?” Participants had to give an answer on a 10-point Likert scale with the additional category “unknown person”.

#### Explicit rating task

In line with the study of Hutcherson et al. [7], the explicit rating task consisted of the following three questions: “1) “How positive do you feel toward the person/object in the photo “, 2) “How similar is the person/object to you?” and 3) “How connected do you feel to the person/object in the photo?” All three questions were asked in a random order on each of the six pictures, which were described in the procedure section (self, stranger, 3 politicians and a lamp), and rated on a 7-point Likert scale from”not at all” to “very much”. A composite score [7] was built for each picture by calculating the mean of the three questions. Internal consistency was determined using McDonalds total omega from the R package psych (version 2.9.1, [25]) and was *ω* = 0.89 for the pre-test and *ω* = 0.85 for the post-test.

#### Implicit affective priming task

**Fig. 2:**
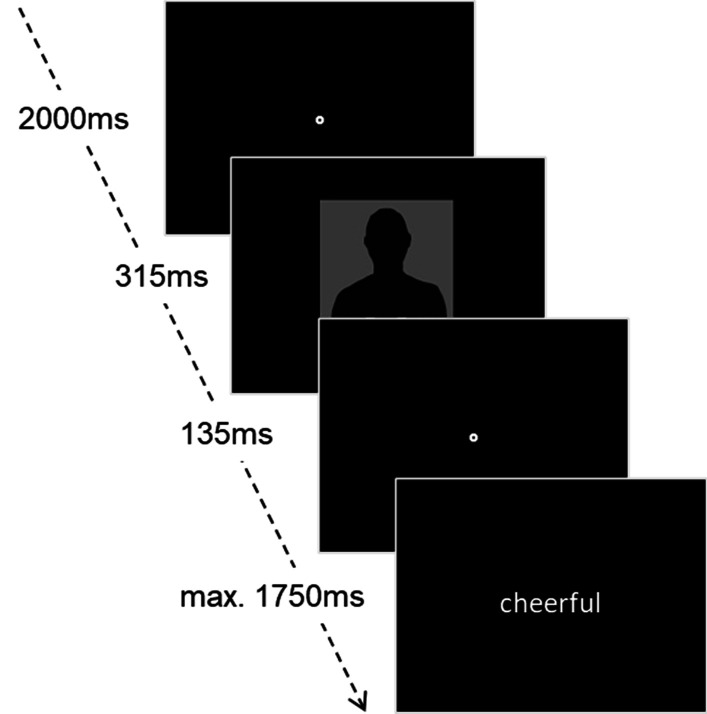
Procedure of the implicit affective priming-task

The implicit task was comprised of an affective priming paradigm [7, 26]. The same six stimuli as in the explicit task were repeated 18 times (9 × paired with a positive word, 9 × paired with a negative word) in a random order, resulting in 108 trials in total [7]. After an initial fixation point on the screen, which was shown for 2000 ms, a picture was presented briefly for 315 ms, followed by another 135 ms fixation point. Afterwards, a word appeared on the screen, picked from a list of 9 negative and 9 positive words, which were retrieved from the Berlin Affective Word List (BAWL-R) [27]. Consequently, each picture was paired with each word once. The participants had to indicate via mouse-click if the word was positive or negative. They had to answer as quickly as possible, otherwise the word disappeared after 1750 ms and the trial was excluded (Fig. 2).

On average, 8.20 (SD = 3.27) trials were excluded per participant due to reaction times above or below two standard deviations from the mean or an reaction time below 100 ms. After removing these outliers and excluding participants with more than 50% missing values or false answers, the remaining mean error rate of M = 13.49, SD = 14.88 (of 216 trials in total) was determined. After checking visually that empty values were missing at random, they were imputed by multiple imputation algorithm and pooling means. Subsequently, the difference between the mean reaction times for the negative words and the mean reaction times for the positive words of the same picture was used as indicator for the implicit attitude toward the respective picture [7]. Therefore, a higher difference score reflected a more positive implicit evaluation. 

#### Positive and negative affect scale (PANAS)

The Positive and Negative Affect Scale (PANAS) had a 5-point Likert scale and consisted of the two main factors positive and negative affect [18]. While it is constructed to measure either trait or state affect, the state version was used in this study. The German version of this questionnaire showed an adequate internal consistency of α = 0.86 and a good internal and external validity [28]. In the present study, we found *ω* = 0.87 (positive) and *ω* = 0.87 (negative) for the pre-test and *ω* = 0.89 (positive) and *ω* = 0.89 (negative) for the post-test.

### Statistical analysis

First, it was analyzed using a Wilcoxon signed-rank test (due to a violation of the normality assumption) if the sympathy rating of the politicians differed significantly between the target politician and the second and third most disliked politicians.

Second, to test if LKM had effects on the mood compared to Imagery, we conducted separate 2 (group) * 2 (time) analyses of variance for positive and negative mood.

Third, to find out if LKM had effects on explicit positivity compared to Imagery, we conducted a 5 (picture: most-, second-, third disliked politician, self, neutral other, object) * 2 (time: baseline, post-test) * 2 (group: LKM, Imagery) repeated measures ANOVA for the explicit evaluation composite score.

Fourth, to analyze if LKM had effects on implicit positivity compared to Imagery, we conducted a 5 (picture: most-, second-, third disliked politician, self, neutral other, object) * 2 (time: baseline, post-test) * 2 (group: LKM, Imagery) repeated measures ANOVA for the implicit evaluation composite score.

It was also analyzed exploratorily in separate analyses, if the effect of the intervention dependent on meditation practice (hours/month) by including it as covariate in the 2 * 2 ANOVAs of mood, explicit and implicit attitudes.

If assumptions were violated, non-parametric alternatives were used. If sphericity was violated, the relevant results were Huynh–Feldt corrected [29]. Analyses were performed using IBM Statistics SPSS 28.

## Results

### Sympathy rating of politicians

Our results showed that the sympathy rating differed significantly between the most disliked politician (*Mdn* = 0) and the second disliked politician (*Mdn* = 0), *Z* =  − 4.61, *p* < 0.001. The difference between the most disliked and the third disliked politician (*Mdn* = 2) was highly significant as well, *Z* =  − 6.20, *p* < 0.001.

### Mood effects

For the positive mood, a “time” effect was observed (Table 1). The positive mood dropped from the baseline (*M* = 3.30, *SD* = 0.63) to the post-test (*M* = 3.14, *SD* = 0.67). There was no “group” effect for the positive mood, as well as no significant interaction between “time” and “group” (Table 1), although a medium effect size was found (LKM pre: *M* = 3.32, *SD* = 0.63; post: *M* = 3.05, *SD* = 0.72; Imagery pre: *M* = 3.27, *SD* = 0.63; post: *M* = 3.22, *SD* = 0.62).

**Table 1:** ANOVA results for the positive and negative subscale of the PANAS

	PANAS positive				PANAS negative			
*Predictor*	*df1, df2*	*F*	*p*	*η*2_*p*_	*df1, df2*	*F*	*p*	*η*2_*p*_
Time	1, 67	7.65	.007	.102	1, 67	6.61	.012	.090
Group	1, 67	0.17	.686	.002	1, 67	1.80	.185	.026
Time*group	1, 67	3.58	.063	.051	1, 67	3.30	.074	.047

For the negative mood, we also observed only a “time” effect (Table 1). The negative mood dropped from the baseline (*M* = 1.55, *SD* = 0.47) to the post-test (*M* = 1.45, *SD* = 0.48). There was no “group” effect for the negative mood, as well as no significant interaction between “time” and “group (Table 1), although a medium effect size was found (LKM pre: *M* = 1.59, *SD* = 0.53; post: *M* = 1.56, *SD* = 0.55; Imagery pre: *M* = 1.51, *SD* = 0.41; post: *M* = 1.35, *SD* = 0.39).

The results of both analyses stayed the same when meditation practice was included.

### Explicit evaluative responses

**Fig. 3:**
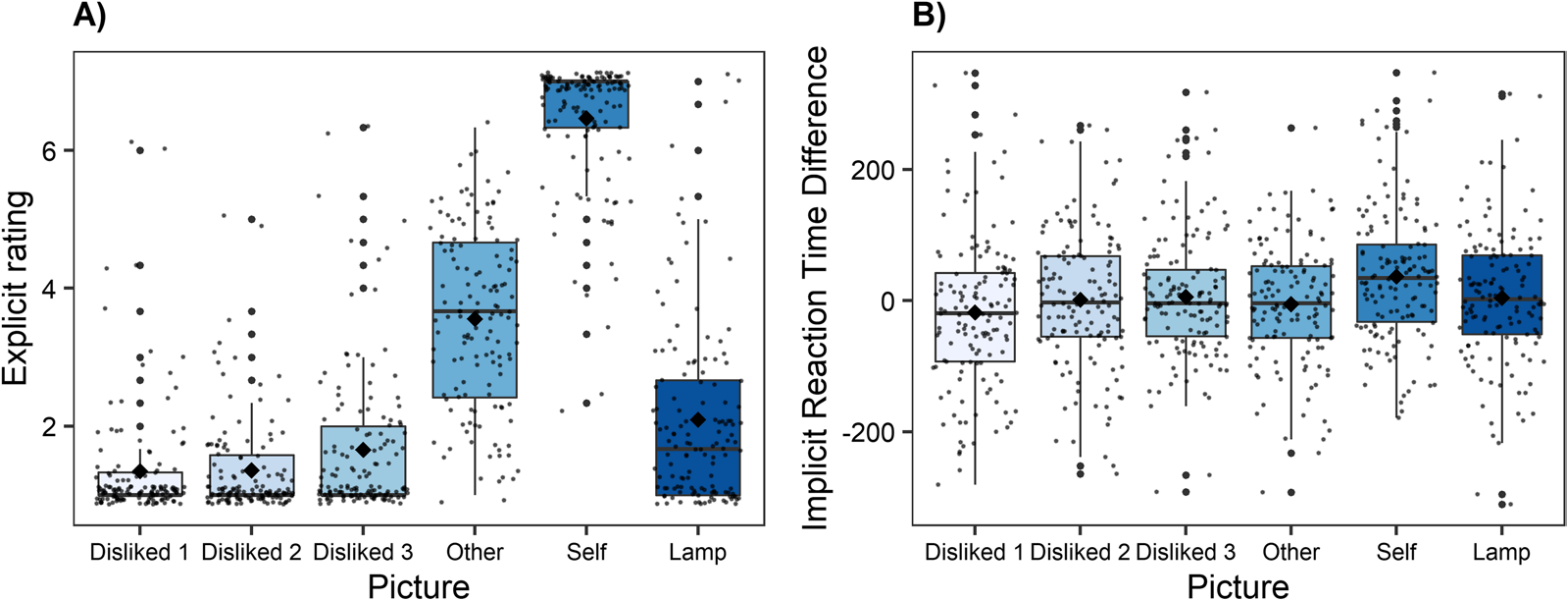
Plot of the main effect of picture on explicit (**A**) and implicit (**B**) attitudes. Means are represented by the diamond shaped dots and medians by the horizontal lines

**Fig. 4:**
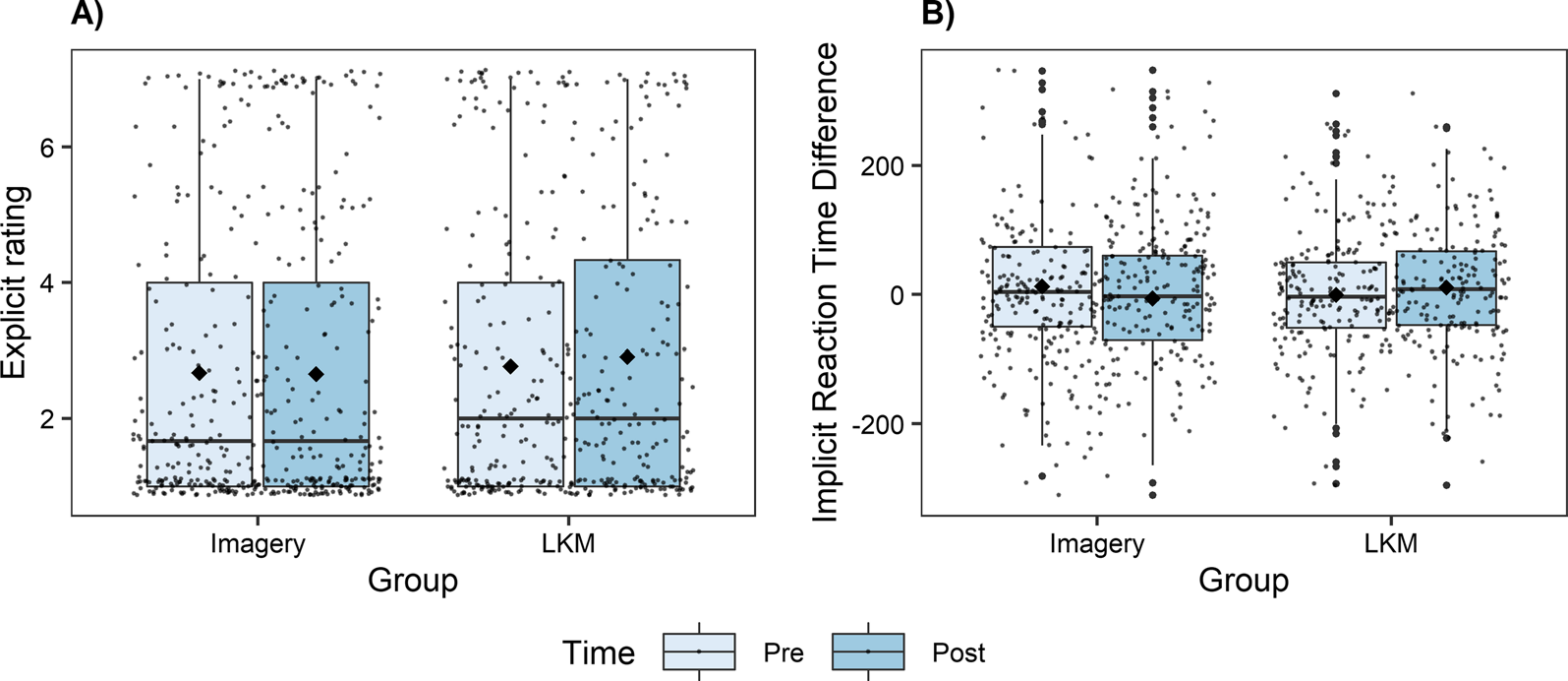
Interaction effect of “Time*Group” on explicit (**A**) and implicit (**B**) attitudes. Means are represented by the diamond shaped dots and medians by the horizontal lines

No main effect of “time” was found regarding the explicit ratings (Table 2). However, a significant main effect of “picture”, but no significant interaction between both factors was found (Table 2). Decomposition of this effect using simple contrasts indicated that the explicit rating between the most disliked politicians did not differ from the rating of the second disliked politician (*p* = 0.926), but from the attitude toward the third disliked politician (*p* = 0.007), the neutral other person (*p* < 0.001), the picture of the self, (*p* < 0.001), and the object (*p* < 0.001), see Table 3, Fig. 3A). There was no main effect of “group”, and neither significant interactions between “group*picture”, “time*group”, nor a significant three-way interaction (Table 2). However, the effect size for the time*group interaction was medium-sized (Fig. 4A), indicating a slight improvement after the LKM meditation. Including the covariate meditation practice did not change the results.

**Table 2:** ANOVA results for explicit and implicit attitudes

	Explicit attitudes				Implicit attitudes			
*Predictor*	*df1, df2*	*F*	*p*	*η*2_*p*_	*df1, df2*	*F*	*p*	*η*2_*p*_
Time	1, 67	2.15	.148	.031	1,67	0.18	.669	.003
Pictures	3.83, 256.35	349.77	< .001	.839	4.83, 323.49	4.76	< .001	.066
Group	1, 67	1.68	.200	.024	1, 67	0.03	.854	.001
Time*picture	4.53, 303.73	1.99	.087	.029	4.97, 333.02	0.91	.473	.013
Time*group	1, 67	3.50	.066	.050	1, 67	3.33	.072	.047
Group*picture	3.83, 256.35	0.51	.719	.008	4.83, 323.49	1.09	.363	.016
Time*group* picture	4.53, 303.73	0.81	.534	.012	4.97, 333.02	0.88	.497	.013

**Table 3:** Means and standard deviations of explicit and implicit ratings in each group

	Explicit				Implicit			
	Control		LKM		Control		LKM	
	M	SD	M	SD	M	SD	M	SD
**Target disliked**								
Pre	1.20	0.60	1.32	0.95	− 5.71	145.17	− 4.67	108.48
Post	1.28	0.57	1.61	1.11	− 43.16	101.08	− 16.88	109.70
**2nd disliked**								
Pre	1.46	0.74	1.29	0.75	15.85	115.81	0.53	101.86
Post	1.30	0.51	1.38	0.80	− 39.85	100.87	31.24	89.41
**3rd disliked**								
Pre	1.74	1.23	1.69	1.13	8.80	104.70	− 0.89	88.12
Post	1.47	0.83	1.75	1.11	− 4.20	97.29	20.57	90.51
**Neutral other**								
Pre	3.45	1.32	3.47	1.07	− 0.45	103.00	− 22.07	78.78
Post	3.55	1.37	3.77	1.31	− 0.09	91.59	0.24	90.25
**Self**								
Pre	6.31	1.13	6.52	0.74	38.62	104.72	21.92	93.88
Post	6.42	1.00	6.63	0.77	51.78	119.93	34.65	86.18
**Lamp**								
Pre	1.86	1.36	2.31	1.19	18.77	97.42	1.66	107.55
Post	1.93	1.33	2.31	1.36	− 0.49	112.21	− 4.03	98.47

### Implicit evaluative responses

There was no significant main effect of “time”, but a significant effect of “picture” (Table 2). Decomposition of this effect using simple contrasts indicated that the implicit rating between the most disliked politicians did not differ from the rating of the second disliked politician (*p* = 0.111), the neutral other person (*p* = 0.264), and the object (*p* = 0.050), but from the implicit attitude toward the picture of the third disliked politician (*p* = 0.041) and the self (*p* < 0.001) (see Table 3, Fig. 3B). There was no main effect of “group”, and no significant interaction effects of “time*group”, “picture*group”, “time*picture” or “time*picture*group” (Table 2). However, the effect size for the “time*group” interaction was medium-sized (Fig. 4B), indicating a slight improvement in implicit attitudes after the LKM and a reduction after the Imagery intervention. Including the covariate meditation practice did not change the results.

## Discussion

Neither the first nor the second hypothesis could be accepted. Our results demonstrated that neither the imagery visualization nor the loving-kindness meditation led to a significant explicit positivity toward disliked politicians, an unknown person, oneself or an object.

In contrast to the study of Hutcherson et al. [7], our results did not demonstrate a significant implicit positivity after a loving-kindness meditation either, although the exact (only translated in German) same loving-kindness meditation and imagery conditions have been used as in the original study. However, p-values indicate a trend for the “time*group” interaction and medium effect sizes were found for this interaction on the dependent variables mood, explicit and implicit attitudes [30]. Means and standard deviations in Table 3 and Fig. 4 point toward a slight improvement in explicit and implicit attitudes for all pictures following the LKM intervention. Because only a trend was found, it cannot be ruled out that differences found in the sample are attributable to random fluctuations in the data. However, it is astonishing that not even a significant time*group effect on the mood of the participants was found, while in the study of Hutcherson et al. [7] the participants’ mood became more positive in the LKM condition, and there was no mood change in the Imagery condition. One reason might be differences in the baseline mood of both samples, but this assumption could not be confirmed due to the missing values in the original study. Besides, the use of the PANAS could be problematic in the scope of this study, since it includes a very broad variety of emotions. A more specific manipulation check for the LKM could be more informative, such as a questionnaire which focuses on prosocial emotions, like the Appreciative Joy Scale from Zeng et al. [31]. Another reason could be the different sample compositions. In the study of Hutcherson et al. [7], the whole sample was more experienced in meditation (1.7 h per month compared to 0.5 h per month). Therefore, we wonder if a short loving-kindness meditation only helps if meditation is more accepted within the sample. However, including meditation practice as covariate did not lead to different results. Furthermore, Seppala et al. [32] demonstrated, in line with the study of Hutcherson et al. [7], that a short loving-kindness meditation of ten minutes can improve well-being and the feelings of connection in a sample of psychology students. However, the students in our study were from the subject of applied movement sciences. Most other studies integrate psychology students as a sample, which might be an important point in the research field of mindfulness, speculating that psychology students are more open to mindfulness interventions. Besides, the study of Hutcherson et al. [7] was conducted in Canada, while ours took place in Europe. Accordingly, cultural effects might be possible.

The findings, that the most disliked politician was rated more negatively than the self, the object, and the unknown person in the explicit task, and also more negatively than the self and the object in the implicit task, imply that the most disliked politician was indeed rated most negatively as indicated by the politician sympathy rating task.

In general, even though we have used the same paradigm as Hutcherson et al. (2008)—only replacing the pictures of the strangers with pictures of politicians—we could not clearly replicate any of the effects of the original study. One possible conclusion would be that there is simply no or a smaller effect, which cannot be reliably detected with our sample size, on the attitude towards disliked politicians as compared to neutral strangers. Besides, 6 min is rather short for an effectful intervention. Possibly, the effectiveness of short interventions depends on other variables that were not studied in our research design, which may explain the trend in our data. Even though the study of Hutcherson et al. shows a different picture, it is questionable whether such a short practice can induce real changes that extent expectancy effects.

### Limitations and future research

The study is limited by the fact that the LKM intervention did not lead to a positive mood augmentation but instead to a drop in positive mood, although after both interventions the negative mood dropped, as well. Accordingly, participants mood became more indifferent and neutral. This finding might also be due to a general fatigue effect evoked by the length of the experiment (approximately 45 min). Furthermore, our pictures of the strangers were not gender matched as in the study of Hutcherson et al. [7]. However, this might be not essential because the attitudes toward strangers were not the target in the present study. Still, gender differences could be relevant in affective priming paradigms [33], as well as in meditation interventions [34].

Although this study does not show the same results as the study of Hutcherson et al., the results are still important: At least, this study shows that a possible influence of a short intervention on emotional evaluations should be treated with caution. The claim that a short loving-kindness meditation enhances social connectedness might awake false hopes. It might be possible and in line with van Dam et al. [9], who state that the hype around meditation studies must be minded, that the possible influence of short bouts of meditation is not more than an artefact of arousal and mood, comparable to the long-lasting debate of the possible enhancing effect of listening to Mozart on spatial intelligence [35]. Just like the music and cognition research, future studies must examine the effects of a long-lasting meditation training on explicit and implicit evaluations of the self and disliked politicians and furthermore investigate the sustainability of those effects. A single intervention effect might either be very small, depend on other variables or it might not be more than an attention effect. Besides, the respective sample, as well as the country, in which short meditation studies are conducted, should be regarded as important points discussing different results.

## Conclusion

With only a few minor modifications, the experimental design of Hutcherson et al. (2008) was used. We did not find the same effects, however both interventions applied here showed an effect on the mood of the participants. It must be concluded that the effect of single short meditation interventions should be regarded with caution to ensure that they do not only evoke some kind of arousal effect.

## Data Availability

The datasets generated and analysed during the current study are available in the OSF repository, https://osf.io/bnf2k/ (https://doi.org/10.17605/OSF.IO/BNF2K).

## Abbreviations

ADFES: Amsterdam Dynamic Facial Expression Set
BAWL-R: Berlin Affective Word List
LKM: Loving-Kindness Meditation
MBSR: Mindfulness-Based Stress Reduction
PANAS: Positive and Negative Affect Scale

## References

[1] Friese M, Smith CT, Koever M, Bluemke M (2016). Implicit measures of attitudes and political voting behavior. Soc Personal Psychol Compass.

[2] Rydell RJ, McConnell AR (2006). Understanding implicit and explicit attitude change: a systems of reasoning analysis. J Pers Soc Psychol.

[3] Lundberg KB, Payne BK (2014). Decisions among the undecided: implicit attitudes predict future voting behavior of undecided voters. PLoS ONE.

[4] Sherman JW, Gawronski B, Trope Y (2014). Dual-Process Theories of the Social Mind.

[5] Greenwald AG, Lai CK (2020). Implicit social cognition. Annu Rev Psychol.

[6] Cameron CD, Brown-Iannuzzi JL, Payne BK (2012). Sequential priming measures of implicit social cognition: a meta-analysis of associations with behavior and explicit attitudes. PSPR.

[7] Hutcherson CA, Seppala EM, Gross JJ (2008). Loving-kindness meditation increases social connectedness. Emotion.

[8] Davidson RJ, Kaszniak AW (2015). Conceptual and methodological issues in research on mindfulness and meditation. Am Psychol.

[9] van Dam NT, van Vugt MK, Vago DR, Schmalzl L, Saron CD, Olendzki A (2018). Mind the hype: a critical evaluation and prescriptive agenda for research on mindfulness and meditation. Perspect Psychol Sci.

[10] Hölzel BK, Lazar SW, Gard T, Schuman-Olivier Z, Vago DR, Ott U (2011). How does mindfulness meditation work? Proposing mechanisms of action from a conceptual and neural perspective. Perspect Psychol Sci.

[11] Matko K, Sedlmeier P (2019). What is meditation? Proposing an empirically derived classification system. Front Psychol.

[12] Dahl CJ, Lutz A, Davidson RJ (2015). Reconstructing and deconstructing the self: cognitive mechanisms in meditation practice. Trends Cogn Sci.

[13] Lippelt DP, Hommel B, Colzato LS (2014). Focused attention, open monitoring and loving kindness meditation: effects on attention, conflict monitoring, and creativity—a review. Front Psychol.

[14] Salzberg S. Lovingkindness: the revolutionary art of happiness. Boston, London: Shambhala; 2018.

[15] Fredrickson BL, Boulton AJ, Firestine AM, van Cappellen P, Algoe SB, Brantley MM (2017). Positive emotion correlates of meditation practice: a comparison of mindfulness meditation and loving-kindness meditation. Mindfulness.

[16] Lumma A-L, Kok BE, Singer T (2015). Is meditation always relaxing? Investigating heart rate, heart rate variability, experienced effort and likeability during training of three types of meditation. Int J Psychophysiol.

[17] Kropp A, Sedlmeier P (2019). What makes mindfulness-based interventions effective? An examination of common components. Mindfulness.

[18] Watson D, Clark LA, Tellegen A (1988). Development and validation of brief measures of positive and negative affect: the PANAS scales. J Pers Soc Psychol.

[19] Norris CJ, Creem D, Hendler R, Kober H (2018). Brief mindfulness meditation improves attention in novices: evidence from ERPs and moderation by neuroticism. Front Hum Neurosci.

[20] Schneider MC, Bos AL (2014). Measuring stereotypes of female politicians. Polit Psychol.

[21] Schneider MC, Bos AL (2011). An exploration of the content of stereotypes of black politicians. Polit Psychol.

[22] Anderson SF, Kelley K, Maxwell SE (2017). Sample-size planning for more accurate statistical power: a method adjusting sample effect sizes for publication bias and uncertainty. Psychol Sci.

[23] Forschungsgruppe Wahlen. Politbarometer. 2019. https://www.forschungsgruppe.de/Umfragen/Politbarometer/Archiv/Politbarometer_2019/Februar_I_2019/. Accessed 3 Mar 2020.

[24] van der Schalk J, Hawk ST, Fischer AH, Doosje BJ (2011). Moving faces, looking places: The Amsterdam Dynamic Facial Expressions Set (ADFES). Emotion.

[25] Revelle W (2021). psych: procedures for psychological, psychometric, and.

[26] Fazio RH, Jackson JR, Dunton DC, Williams CJ (1995). Variability in automatic activation as an unobtrusive measure of racial attitudes: A bona fide pipeline?. J Pers Soc Psychol.

[27] Võ MLH, Conrad M, Kuchinke L, Urton K, Hofmann MJ, Jacobs AM (2009). The Berlin Affective Word List Reloaded (BAWL-R). Behav Res Methods.

[28] Breyer B, Bluemke M. Deutsche Version der Positive and Negative Affect Schedule PANAS (GESIS Panel). Mannheim, Germany: GESIS Leibniz-Institut für Sozialwissenschaften; 2016.

[29] Huynh H, Feldt LS (1976). Estimation of the box correction for degrees of freedom from sample data in randomized block and split-plot designs. J Educ Behav Stat.

[30] Amrhein V, Greenland S, McShane B (2019). Scientists rise up against statistical significance. Nature.

[31] Zeng X, Liao R, Zhang R, Oei TPS, Yao Z, Leung FYK, Liu X (2017). Development of the appreciative joy scale. Mindfulness.

[32] Seppala EM, Hutcherson CA, Nguyen DTH, Doty JR, Gross JJ (2014). Loving-kindness meditation: a tool to improve healthcare provider compassion, resilience, and patient care. J Compassionate Health Care.

[33] Donges U-S, Kersting A, Suslow T (2012). Women’s greater ability to perceive happy facial emotion automatically: gender differences in affective priming. PLoS ONE.

[34] Rojiani R, Santoyo JF, Rahrig H, Roth HD, Britton WB (2017). Women benefit more than men in response to college-based meditation training. Front Psychol.

[35] Thompson WF, Schellenberg EG, Husain G (2001). Arousal, mood, and the Mozart effect. Psychol Sci.

